# Supplementary material for: Multi-target and ultra-high-speed optical wireless communication using a thin-film lithium niobate optical phased array
Source: Nat Commun. 2025 Dec 15;17:969. doi: 10.1038/s41467-025-67696-3 (PMC12847847; doi:10.1038/s41467-025-67696-3)
Supplement: Supplementary file 1 — Supplementary Information [file 41467_2025_67696_MOESM1_ESM.pdf]

Supplementary information for

Multi-target and ultra-high-speed optical wireless communication using a thin-film lithium niobate optical phased array

Xiaoyue Ma[1,†], Mingrui Yuan[1,†], Jingchi Li[2], Hongdong Zhang[1], Baichuan He[1], Pu Zhang[1], Yongheng Jiang[1], Huifu Xiao[1], Guanghui Ren[3], Arnan Mitchell[3], Yikai Su[2] & Yonghui Tian[1,4,*]

[1] School of Physical Science and Technology, Lanzhou University, Lanzhou 730000, Gansu, China.

[2] State Key Laboratory of Photonics and Communications, School of Information and Electronic Engineering, Shanghai Jiao Tong University, Shanghai, 200240, China.

[3] Integrated Photonics and Applications Centre (InPAC), School of Engineering, RMIT University, Melbourne, VIC 3001, Australia.

[4] School of Mathematics and Physics, North China Electric Power University, Beijing 102206, China

[†]These authors contributed equally to this work.

Corresponding author: *tianyh@lzu.edu.cn.

This supplementary information file includes:

Supplementary Note 1: Manufacturing process.

Supplementary Note 2: Basic principles of OPA.

Supplementary Note 3: Simulation of trapezoidal plate antenna.

Supplementary Note 4: Simulation and design of passive components.

Supplementary Note 5: OPA far-field beam spot calibration.

Supplementary Note 6: Far-field beam characterization setup for OPA.

Supplementary Note 7: Power consumption principle and calculation.

Supplementary Note 8: OPA working distance and stability measurement device.

Supplementary Note 9: DSP process description for 320 Gbps 16 QAM transmission.

Supplementary Note 10: Performance scaling of OPA.

Supplementary Table 1: TFLN OPA system performance comparison.

Supplementary Note 1: Manufacturing process.

Figure S1 illustrates the fabrication process of an optical phased array (OPA) on a lithium niobate on insulator (LNOI) platform. A photoresist mask is first spin-coated onto the lithium niobate (LN) layer. Waveguide patterning is performed using deep ultraviolet (DUV) lithography, followed by dry etching to transfer the pattern into the LN layer. After the photoresist is removed, a silicon dioxide (SiO_2) cladding layer is subsequently deposited. A gold layer is deposited and etched to form electrodes. Finally, the OPA chip is fabricated. The waveguide sidewalls exhibit an inclination angle of approximately 67°.

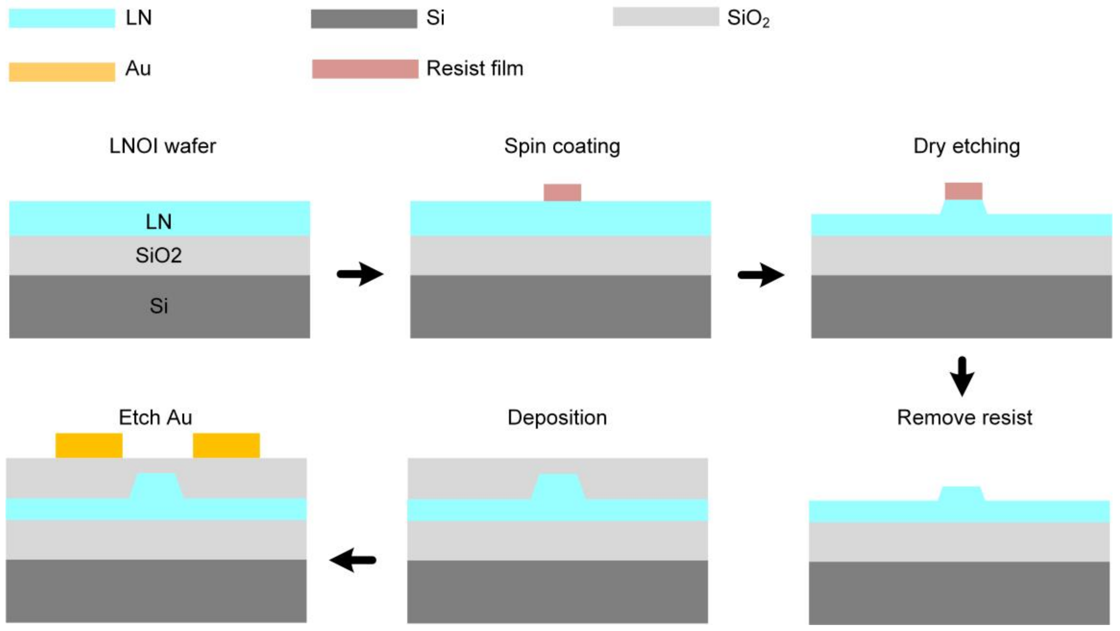

Figure S1 The fabrication process of an OPA on an LNOI platform

Electrically, the system uses a 2D packaging architecture with wire bonding to connect the chip electrodes to a printed circuit board (PCB). A double-row pin header with 2.54 mm pitch offers a standardized plug-and-play interface for integration into external drive control systems. Optically, a polarization-maintaining fiber array is employed. It has a mode field diameter of 3.2 μm at 1550 nm wavelength to ensure efficient coupling into the chip's waveguides. The polarization axis of the fiber aligns with the transverse electric (TE) mode of the waveguides, which minimizes mode mismatch and polarization-dependent loss. The optical interface between the fiber and the chip is stabilized via precise alignment and permanent adhesive fixation, improving

long-term reliability. Output fiber arrays are terminated with FC/APC connectors, yielding a total packaged loss of 4.7 dB while maintaining low insertion loss and reflectivity.

Supplementary Note 2: Basic principles of OPA.

Figure S2 schematically illustrates the operational principle of the OPA. The detection plane is positioned at a distance z_0 from the emitter array. For a specific point $P(z_0, x_1)$ on this plane, where the observation distance z_0 is much greater than x_1 ($z_0 >> x_1$), the following condition is satisfied:

$$\sin\theta \approx \tan\theta = \frac{x_1}{z_0} \tag{S1}$$

To meet the Fraunhofer diffraction condition ($z_0 > 2Nd/\lambda$), where N represents the number of channels, the emitter aperture is approximated as a rectangular source. This approximation allows the near-field electric field intensity to be derived as:

$$e(x) = \sum_{n=0}^{N-1} rect\left(\frac{x-nd}{a}\right) e^{jn\varphi} \tag{S2}$$

The far-field electric field distribution corresponds to the Fourier transform of the near-field electric field. Thus, the far-field electric field at point P is expressed as:

$$e(x_1) = a\,\mathrm{sinc}(\alpha)\left[\frac{\sin\frac{N(\chi-\varphi)}{2}}{\sin\frac{\chi-\varphi}{2}}\right] e^{j\left[(N-1)\frac{\chi-\varphi}{2}\right]} \tag{S3}$$

Therefore, the optical intensity is given by:

$$I(x_1) = |e(x_1)|^2 = e(x_1)\big(e(x_1)\big)^* = I_0\left(\frac{\sin\alpha}{\alpha}\right)\left[\frac{\sin\frac{N(\chi-\varphi)}{2}}{\sin\frac{\chi-\varphi}{2}}\right]^2 \tag{S4}$$

where: $\chi = kd\sin\theta$, $\alpha = \frac{1}{2}ka\sin\theta$, $I_0 = a^2$, with a denotes the emitter aperture width, $(e(x_1))^*$ represents the complex conjugate operation applied to the electric field, and the wavenumber $k = 2\pi/\lambda$. It follows that:

$$\sin\theta = \frac{\lambda_0 \Delta\phi}{2\pi d} \tag{S5}$$

where λ_0 is the wavelength, $\Delta\phi$ is the phase shift, and d is the waveguide spacing. Therefore, the waveguide spacing must be half the wavelength ($d \leq \lambda_0/2$), when the steering angle of the phase direction is at its maximum.

Wavelength steering angle control is achieved through wavelength tuning. The output coupling angle is determined by the grating equation and can be expressed as:

$$\sin\varphi = \frac{\Lambda n_{eff} - \lambda_0}{n_0 \Lambda} \tag{S6}$$

where Λ is the antenna period, n_{eff} is the effective refractive index of the waveguide mode, and n_0 is the background refractive index. The steering angle in the wavelength direction is highly dependent on the antenna design. A larger contrast in the effective refractive index results in a larger steering angle.

The horizontal and vertical resolutions of the OPA are governed by distinct parameters. The horizontal beam resolution, characterized by its full width at half maximum (FWHM), is primarily determined by the overall aperture size, which is the product of the number of antenna elements (N) and their pitch (d). According to the relation $\theta_{\mathrm{FWHM}} = \frac{0.886\lambda}{Nd\cos\theta}$, a larger aperture yields a narrower beam, thus enhancing the horizontal resolution. Conversely, the vertical beam resolution is chiefly dictated by the radiation properties of the trapezoidal grating antennas. The effective grating length sets the vertical beam divergence.

The sidelobe suppression ratio (SLSR), $\mathrm{SLSR} = 10\log\left(\frac{I_{\mathrm{sidelobe}}}{I_{\mathrm{mainlobe}}}\right)$, defined as the ratio of the mainlobe peak intensity to the highest sidelobe peak intensity, is a key parameter in the system. Enhancing the SLSR improves power concentration within the mainlobe, which increases both the scanning distance and the scanning accuracy of the mainlobe peak.

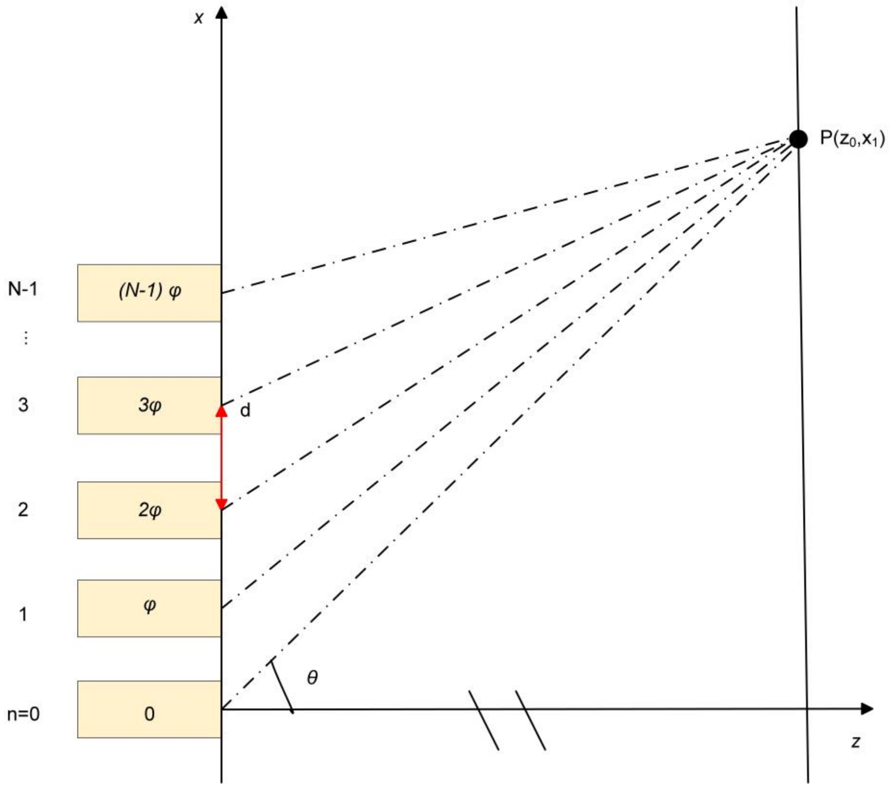

Figure S2 Far-field diffraction pattern of the phase-tuned array aperture.

Supplementary Note 3: Simulation of trapezoidal plate antenna.

In OPA design, the waveguide array and antenna configuration are particularly critical since they directly determine the system's beam steering performance and signal integrity. When light from single-mode waveguides couples into the antenna, near-field free diffraction occurs at the terminus of the waveguide array. Provided that strict phase coherence is maintained at the output of the waveguide array, this diffraction produces a quasi-planar wavefront in the optical field at the antenna interface. The near-field propagation angle (χ) of the wavefront depends on optical interference within the waveguide array. By adjusting the phase shift between adjacent waveguides, the far-field steering angle (θ) of the antenna can be correspondingly controlled. The distribution of diffraction order efficiencies, such as the first-order diffraction efficiency and sidelobe suppression level, depends on the structural parameters of the grating antenna. These parameters include tilt angle, period, and duty cycle. Simulation and optimization of these parameters are essential to effectively suppress reflection and stray light, which is crucial for maintaining high signal quality in OPA systems.

To achieve a balance between low reflection and high transmission efficiency, this study uses a slab grating antenna with a trapezoidal profile. Figure S3a shows the structure of this trapezoidal-profile slab grating antenna, offering a visual reference for understanding its design features. The tilt angle (γ) of the optimized grating antenna exceeds the in-plane divergence angle of the fundamental waveguide mode. This design reduces Fresnel reflection from the grating sidewalls. Lower sidewall reflection decreases scattering loss, enabling highly efficient and low-distortion free-space forward transmission, which is essential for preserving signal strength in OPA-based communication. The optimized grating antenna has a tilt angle of 20°, a period of 0.8 μm, and a duty cycle of 0.3. Far-field simulations are conducted at wavelengths of 1530 nm, 1550 nm, and 1565 nm using the optimized antenna parameters. Figure S3b displays the resulting far-field profiles, with the aim of verifying the antenna's sidelobe suppression performance across these wavelengths. The profiles clearly show a high SLSR. This is important because it reduces interference from unwanted sidelobes and ensures that most optical power is concentrated in the main beam for effective

communication.

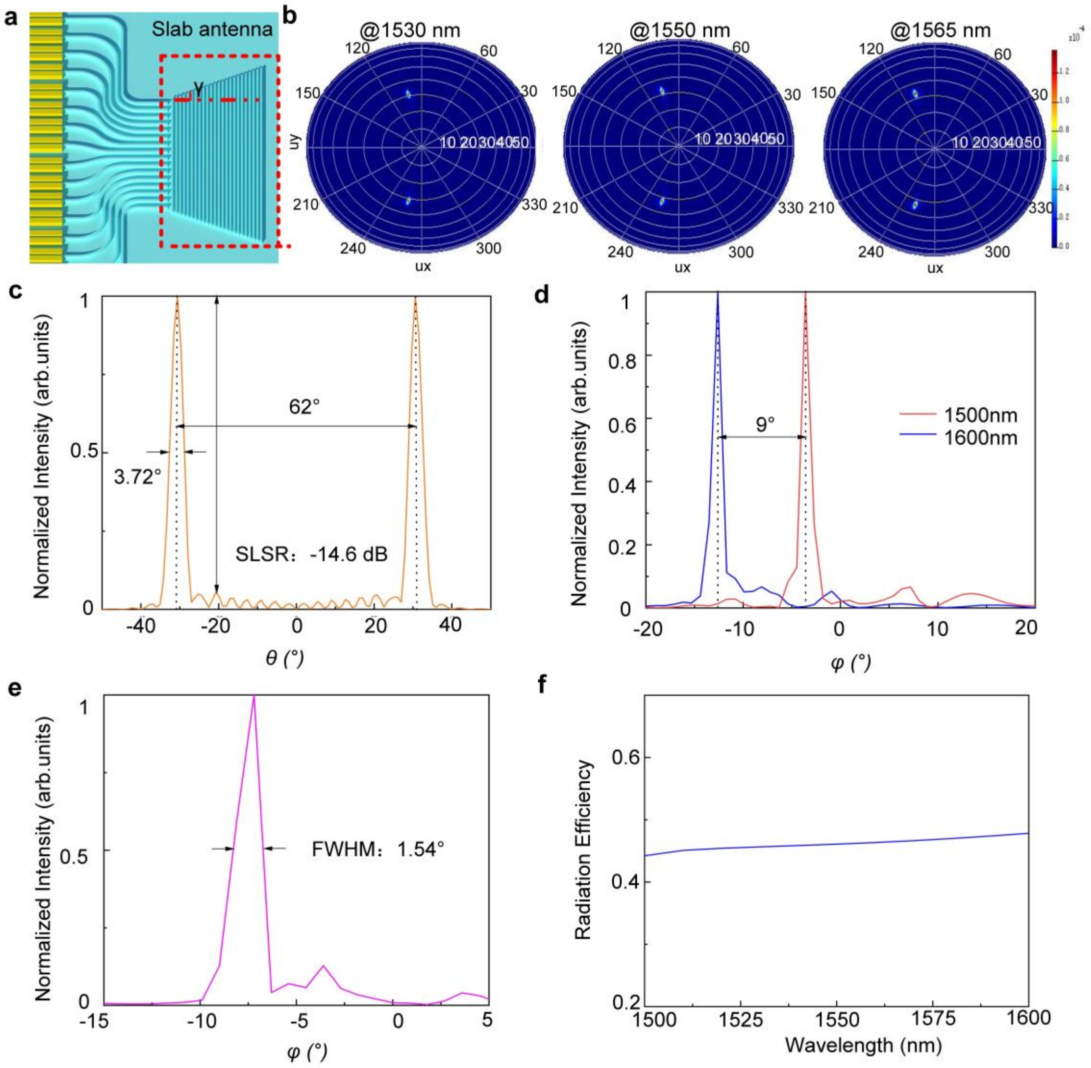

Figure S3 **a** Schematic diagram of a trapezoidal plate grating antenna, where γ is the tilt angle. **b** Simulated far-field intensity distribution of the antenna at 1530 nm, 1550 nm, and 1565 nm. **c** Simulated far-field intensity distribution at the maximum steering angle along the phase direction. **d** Simulated far-field intensity distribution along the wavelength direction. **e** Magnified view of the simulated far-field beam at 1550 nm. **f** The upward radiation efficiency of the simulated OPA antenna at wavelengths of 1500-1600 nm.

Figure S3c analyzes the simulated far-field intensity distribution at the maximum steering angle along the phase direction after optimizing the trapezoidal grating antenna. This analysis verifies whether scattering loss and mode disturbance caused by sidewall reflection are effectively suppressed. The results indicate that at a tilt angle of 20°, both scattering loss and mode disturbance due to sidewall reflection are well suppressed.

With a waveguide spacing of 1.5 μm, the steering angle in the phase direction reaches 62°. This steering angle is accompanied by a side-mode rejection ratio of −14.6 dB and a FWHM of 3.72°. The large steering angle satisfies the OPA's requirement for wide beam coverage, while the high side-mode rejection ratio and narrow FWHM ensure that the main beam remains focused and free from significant interference.

Figure S3d shows the simulated far-field intensity distribution along the wavelength direction for the same trapezoidal grating antenna. Its purpose is to evaluate the antenna's far-field performance in the wavelength direction and verify the stability of beam steering over a broad wavelength range. Across the wavelength range from 1500 nm to 1600 nm, the beam-steering angle in the wavelength direction is 11°, with a corresponding wavelength steering efficiency of −0.09/nm. This high efficiency indicates that the beam direction angle changes greatly with wavelength, which is crucial for maintaining the large angle transmission of the signal across the communication band. The current vertical beam steering range of 11° is mainly limited by the spectral response of the bandwidth of passive devices and the wavelength tuning range of the external laser. Future designs could substantially extend this range by employing apodized or chirped gratings to increase radiation bandwidth [1,2], and exploiting polarization diversity [3,4] or multi-period [5] OPA in the thin-film lithium niobate (TFLN) platform. These strategies, combined with the integration of widely tunable on-chip lasers, are expected to expand the vertical field-of-view (FOV) well beyond 20°, enabling more flexible beam steering for next-generation optical wireless communication (OWC) systems.

Figure S3e offers a magnified view of the simulated far-field beam at 1550 nm. In this magnified view, the FWHM in the wavelength direction is 1.54°. A narrow FWHM in this direction enhances the beam's directionality, improving the accuracy of signal delivery in OPA communication systems. FDTD simulation results show that our design features a radiation efficiency of 44.2%-48% within the 1500nm-1600nm, as shown in Figure S3f.

Supplementary Note 4: Simulation and Design of Passive Components.

To characterize the core components of the OPA system, we first analyze the performance of the 1×2 beam splitter, which serves as a fundamental element for signal distribution. This section uses supplementary figures to present the splitter's structure, optical field characteristics, and broadband loss performance. Figure S4a shows a schematic of the 1×2 beam splitter. This figure clarifies the design basis of the splitter, which uses a multimode interferometer (MMI) coupler. Figure S4b displays the optical field distribution inside the 1×2 MMI coupler at 1550 nm. Figure S4c shows the spectral response of the insertion loss. Its purpose is to quantify the beam splitter's loss performance across a broad wavelength range. At 1550 nm, the splitter achieves a low loss of about 0.02 dB. Over the 1500–1600 nm wavelength range, the loss remains below 0.05 dB. This result indicates excellent broadband performance, which is critical for meeting the OPA's requirement for stable signal transmission across the communication band.

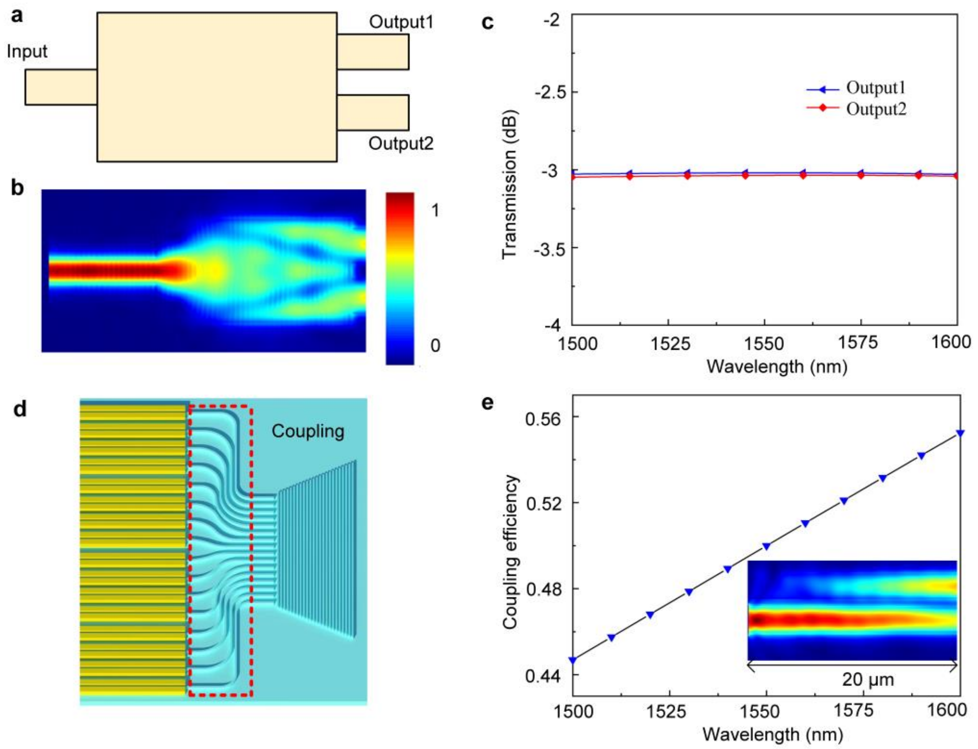

Figure S4 **a** Schematic of the MMI structure. **b** Simulated optical field in the 1×2 MMI coupler at 1550 nm. **c** Simulated insertion loss across 1500–1600 nm. **d** Layout of the bent waveguide coupling structure. **e** Simulated crosstalk in straight waveguide coupling.

In OPAs, the antenna section requires a small element pitch to achieve a wide beam steering range. However, the phase shifter section usually requires a larger waveguide pitch to accommodate driving electrodes. This pitch mismatch creates a need for a transition structure between the phase shifter and the optical antenna. This structure must have low crosstalk and low loss to ensure efficient signal coupling. Crosstalk from adjacent waveguide coupling can significantly degrade antenna performance, so suppressing inter-channel crosstalk is a key design goal. To address this issue, the design uses curved waveguides for coupling.

Figure S4d provides a schematic of the arrayed waveguide structure. This figure illustrates the specific layout of the curved waveguide coupling structure, which is central to the transition design. The schematic clearly shows how the curved waveguides connect the phase shifter and antenna sections, offering a visual reference for the coupling scheme. Figure S4e shows the simulated crosstalk in straight waveguide coupling. This figure highlights the need for the curved waveguide design by comparing it with the straight waveguide performance. Straight waveguides with 1.5 μm center-to-center spacing produce strong crosstalk within a 20 μm propagation distance. This strong crosstalk results from optical coupling between neighboring channels and would impair the OPA's beam steering accuracy. This result confirms that straight waveguides are unsuitable for the transition structure and emphasizes the advantage of the curved waveguide design.

The bend radius of the transition structure directly affects both the compactness and signal integrity of the OPA. The design achieves a minimum bend radius of 80 μm. Figure S5a shows the simulated optical path for an 80 μm radius bend. This figure focuses on light propagation behavior at the minimum bend radius, a critical parameter for the OPA's overall size. The optical path confirms that light propagates through the 80 μm radius bend without significant distortion, supporting the feasibility of the compact design. Figure S5b presents the insertion loss for different bend radii. This figure assesses how the bend radius influences the signal loss of the transition structure. Three bend radii (80 μm, 100 μm, and 120 μm) are simulated and optimized. Over a 100 nm wavelength range, the insertion loss remains below 0.25 dB for all three radii.

The coupling length between adjacent waveguides is another key factor for crosstalk control. In this design, the coupling length is defined as the region where the waveguide spacing is less than 2 μm. To maximize crosstalk suppression, the bend radii are designed to increase from the edges to the center of the array. Figure S5c shows the optical propagation simulation between waveguides with different bend radii. This figure illustrates the light coupling behavior between an 80 μm radius bend and waveguides with 180 μm and 240 μm radii. Only a small amount of light couples into the adjacent waveguide, demonstrating that the varying bend radii effectively reduce unwanted light leakage. Figure S5d shows the simulated crosstalk between waveguides with different bend radii. This figure quantifies how radius differences help suppress crosstalk. Simulations show that when the bend radius difference between adjacent waveguides exceeds 100 μm, crosstalk can be suppressed below –15 dB. This level of suppression meets the OPA's requirement for independent signal transmission between channels, ensuring that beam steering performance is not compromised by inter-channel interference.

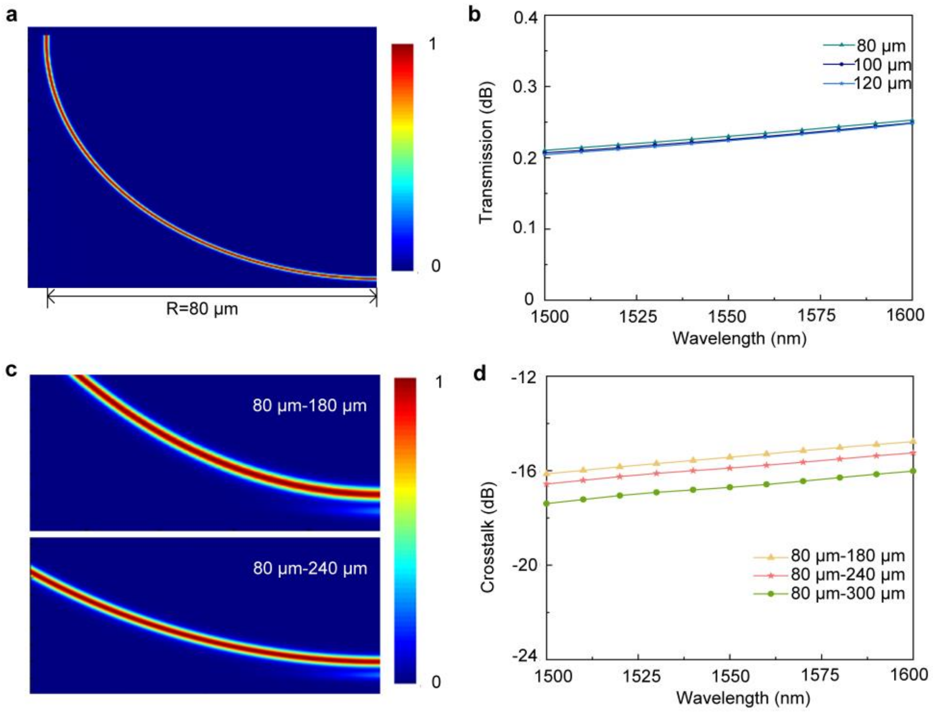

Figure S5 a Simulated optical path for an 80 μm radius bend. **b** Insertion loss for different bend radii. **c** Optical propagation simulation between waveguides with different bend radii. **d** Simulated crosstalk between waveguides of different bend radii.

Supplementary Note 5: OPA far-field beam spot calibration.

Systematic phase errors are introduced into the antenna during OPA manufacturing. These errors result from waveguide sidewall roughness and limited etching precision, which degrade far-field sidelobe performance and reduce beam steering efficiency. Phase calibration is therefore necessary for the OPA. The stochastic parallel gradient descent (SPGD) algorithm is widely used in adaptive optical systems. It adjusts wavefront correctors to compensate for beam wavefront aberrations. In OPA phase noise compensation, the SPGD algorithm controls the voltage of each channel. It performs model-free gradient optimization in multi-channel systems through stochastic parallel perturbation and differential measurement. The SPGD algorithm offers a balance among real-time operation, hardware complexity, and control accuracy in OPA systems, making it a core method for wavefront correction and beam steering. The SPGD algorithm is implemented through a defined procedure. First, a system performance metric is defined as the optimization objective. This metric depends on the control signals applied to the OPA phase shifter. Stochastic perturbation voltages are then applied to each phase shifter channel via the drive circuit, following a Bernoulli distribution.

Figure S6a illustrates the SPGD-based calibration system, offering a visual overview of the calibration workflow. The system is implemented in LabVIEW-NI. A 1550 nm laser output is delivered through a polarization controller (PC) into the OPA chip. The far-field pattern is captured by a far-field measurement setup and transmitted to the LabVIEW control platform on the host computer. Using the SPGD algorithm, the LabVIEW platform iteratively optimizes output voltages sent to a multichannel voltage source field-programmable gate array (FPGA). These voltages drive the phase calibration of the OPA phase shifter array and achieve far-field pattern correction. The clear layout in Figure S6a ensures the reproducibility of the calibration process, which is essential for validating the algorithm. Figure S6b shows the far-field pattern in the initial uncalibrated state, demonstrating the performance limitations of the OPA before calibration. As shown, no distinct main lobe is visible and the intensity is scattered. This distribution leads to dispersed signal strength, which is inadequate for effective optical

communication. The result underscores the necessity of phase calibration to improve beam quality. After calibration using a 16-channel voltage source and the host computer, the far-field pattern at 0° is recorded. Figure S6c displays this post-calibration result, confirming the improvement in beam quality after phase alignment. As shown, the optical power is concentrated at the 0° position. This concentration ensures high main lobe signal integrity, which is critical for stable signal transmission. It also demonstrates that the SPGD algorithm effectively compensates for systematic phase errors, establishing a foundation for reliable beam steering performance in the OPA.

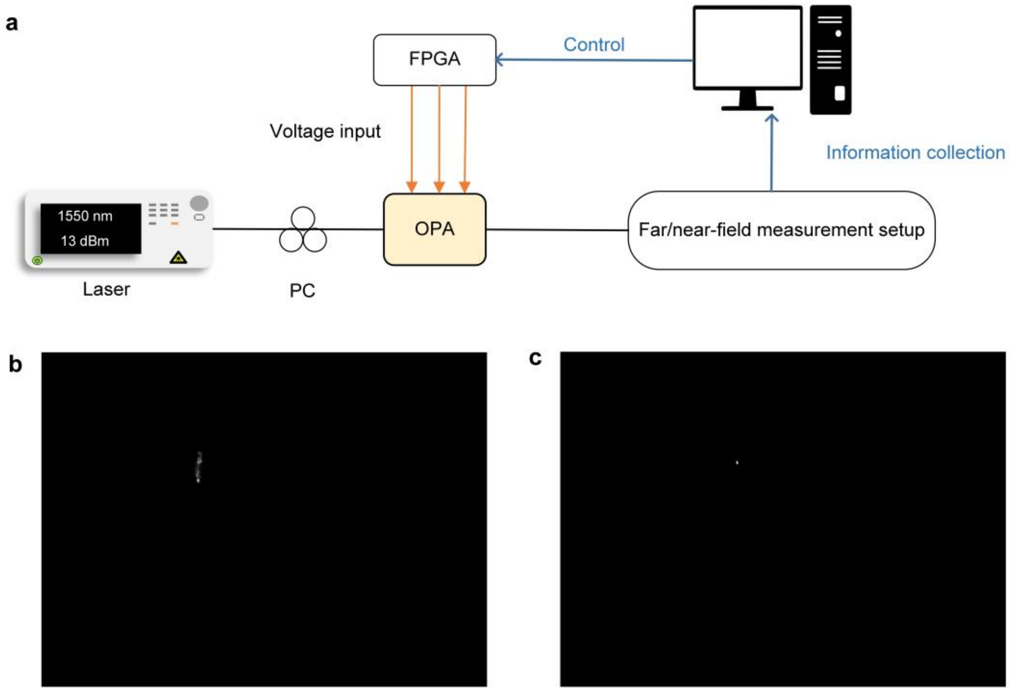

Figure S6 **a** Test system for phase calibration that utilizes the SPGD algorithm implemented in NI LabVIEW. **b** The initial far-field image is captured by the camera. **c** The calibrated far-field image.

SPGD is a blind optimization algorithm designed for multidimensional unconstrained optimization problems. The problem under consideration is formulated as:

$$\max_{u} J(u) \tag{S7}$$

where $J(u)$ denotes the system performance index function to be optimized, and $u=[u_1,u_2,\ldots,u_N]^T$ represents the multidimensional input variable. The objective is to maximize $J(u)$ by adjusting each component of the vector u.

An initial value of the vector u is chosen. A perturbation vector δu is defined such

that it satisfies:

$$\begin{cases} \delta u \sim N(0,\sigma^2 I) \\ E[\delta u]=0 \\ E[\delta u \delta u^T]=\sigma^2 I \end{cases} \tag{S8}$$

That is, the perturbation has fixed variance, zero mean, and isotropic properties. It is randomly generated in each iteration.

Perturbations δu are applied simultaneously to all elements of the vector at the operating point u. Taylor expansions are performed in both positive and negative directions:

$$\begin{cases} J(u+\delta u)=J(u)+\delta u^T \nabla J(u)+\frac{1}{2}\delta u^T H(u)\delta u+o\left(\|\delta u\|^3\right) \\ J(u-\delta u)=J(u)-\delta u^T \nabla J(u)+\frac{1}{2}\delta u^T H(u)\delta u+o\left(\|\delta u\|^3\right) \end{cases} \tag{S9}$$

where:

$$H(u)=\nabla^2 J(u)=\begin{bmatrix} \frac{\partial^2 J}{\partial u_1{}^2} & \frac{\partial^2 J}{\partial u_1 \partial u_2} & \cdots & \frac{\partial^2 J}{\partial u_1 \partial u_N} \\ \frac{\partial^2 J}{\partial u_2 \partial u_1} & \frac{\partial^2 J}{\partial u_2{}^2} & & \frac{\partial^2 J}{\partial u_2 \partial u_N} \\ \vdots & & \ddots & \vdots \\ \frac{\partial^2 J}{\partial u_N \partial u_1} & \frac{\partial^2 J}{\partial u_N \partial u_2} & \cdots & \frac{\partial^2 J}{\partial u_N{}^2} \end{bmatrix} \tag{S10}$$

The difference in the system performance index under bilateral perturbations is calculated as:

$$J_+ - J_- = [J(u+\delta u)-J(u)]-[J(u)-J(u-\delta u)]=J(u+\delta u)-J(u-\delta u) \tag{S11}$$

Applying Taylor expansion to the above expression yields:

$$J_+ - J_- = \left[\delta u^T \nabla J(u)+\frac{1}{2}\delta u^T H(u)\delta u\right] - \left[-\delta u^T \nabla J(u)+\frac{1}{2}\delta u^T H(u)\delta u\right] + o\left(\|\delta u\|^3\right) \tag{S12}$$

By ignoring third-order and higher infinitesimal terms, the expression simplifies to:

$$J_+ - J_- \approx 2\delta u^T \nabla J(u) \tag{S13}$$

Since the gradient $\nabla J(u)$ cannot be directly computed, both sides of equation (S13) are multiplied by δu and the expectation is taken:

$$E[(J_+ - J_-)\delta u] \approx E\left[2\left(\delta u^T \nabla J(u)\right)\delta u\right] \tag{S14}$$

Using the properties of stated in equation (S8), the right-hand side expands to:

$$E\left[2\left(\delta u^T \nabla J(u)\right)\delta u\right] = E[2\delta u \delta u^T]\nabla J(u)=2\sigma^2 \nabla J \tag{S15}$$

yielding:

$$E[(J_+\text{-}J_-)\delta u]\approx 2\sigma^2\nabla J \quad (S16)$$

The gradient estimate is therefore:

$$\nabla J\approx\frac{1}{2\sigma^2}E[(J_+\text{-}J_-)\delta u] \quad (S17)$$

In practice, the expectation cannot be computed exactly. Instead, a single perturbation sample is used to approximate the gradient estimator:

$$\hat{g}=\frac{J_+\text{-}J_-}{2\sigma^2}\delta u \quad (S18)$$

This estimation is affected by third-order and higher errors. If unilateral perturbation is employed, the computational load can be reduced, leading to:

$$\hat{g}=\frac{J_+\text{-}J_-}{\sigma^2}\delta u \quad (S19)$$

However, in this case, the second-order error term $\frac{1}{2}\delta u^T H(u)\delta u$ remains, which reduces the accuracy of the algorithm.

Substituting into the iterative update rule of the conventional gradient descent algorithm:

$$u_j^{n+1}=u_j^n\text{-}\gamma J_j^{'} \quad (S20)$$

yields the SPGD iterative update rule:

$$u_j^{n+1}=u_j^n\text{-}\mu\delta J^n\delta u_j^n \quad (S21)$$

where j=1, …, N is the number of elements, n= 0, 1, … is the iteration index, and $\mu=\frac{\gamma}{2\sigma^2}$ is a constant.

Validation of the SPGD Algorithm

To verify the rationality of the update rule, substitute the Taylor-expanded expression into equation (S19):

$$\hat{g}=\frac{\delta u^T\nabla J(u)}{\sigma^2}\delta u+o\left(\frac{\|\delta u\|^3}{\sigma^2}\right)\delta u \quad (S22)$$

Taking the expectation:

$$E[\hat{g}]=\frac{1}{\sigma^2}E\left[\left(\delta u^T\nabla J(u)\right)\delta u\right]+E\left[o\left(\frac{\|\delta u\|^3}{\sigma^2}\right)\delta u\right] \quad (S23)$$

Based on the isotropic property of the random perturbation:

$$E[\delta u \delta u^T]=\sigma^2 I \tag{S24}$$

Thus:

$$E\left[\left(\delta u^T \nabla J(u)\right)\delta u\right]=E[\delta u^T \nabla J(u)\delta u]=\sigma^2 \nabla J(u) \tag{S25}$$

Higher-order terms in equation (S24):

$$E\left[o\left(\frac{||\delta u||^3}{\sigma^2}\right)\delta u\right]=o(\sigma) \tag{S26}$$

The expectation of the gradient estimate is:

$$E[\hat{g}]=\nabla J(u)+o(\sigma) \tag{S27}$$

Therefore, when condition $\sigma \to 0$ is satisfied, the gradient estimate is asymptotically unbiased. By the consistency of expectation, the iteration direction aligns with the true gradient, which cannot be directly computed.

Summary of the SPGD Algorithm Steps

The steps of SPGD algorithm can be summarized as follows:

(1) Initialize parameters: initial vector $u^{(0)}$, gain coefficient η, perturbation amplitude σ, and maximum iteration count T. Compute the initial performance index J_0.

(2) Generate a Bernoulli-distributed perturbation vector $\delta u_i^{(k)}=\pm\sigma$ at the k-th iteration.

(3) Compute the change in the performance index under symmetric bilateral perturbations: $J_+=J(u^{(k)}+\delta u^{(k)})$ and $J_-=J(u^{(k)}-\delta u^{(k)})$.

(4) Estimate the gradient: $\hat{g}^{(k)}=\frac{J_+-J_-}{2\sigma^2}\delta u^{(k)}$.

(5) Update parameters along the estimated gradient direction: $u_j^{n+1}=u_j^n-\mu\delta J^n \delta u_j^n$.

Steps 2 to 5 are repeated until either the maximum iteration count is reached or the performance metric meets the predefined threshold. The basic flowchart of the SPGD algorithm is shown in Figure S7.

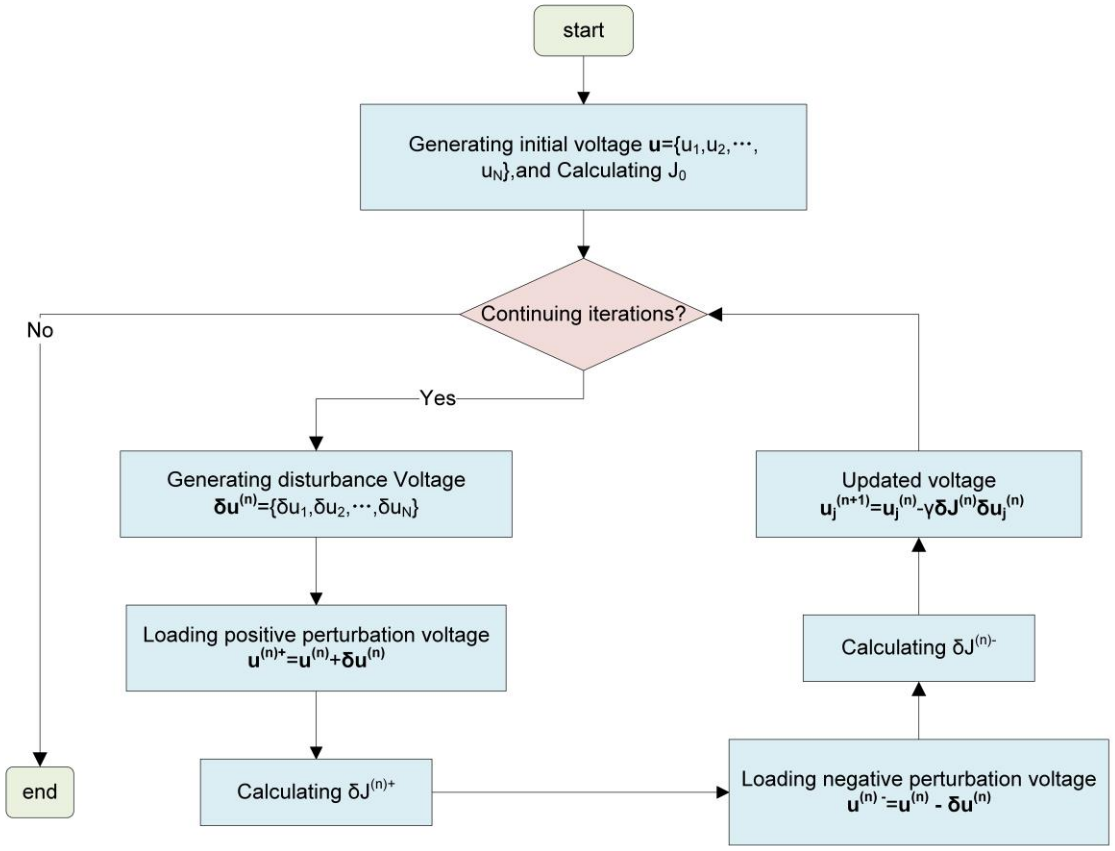

Figure S7 Flow chart of the SPGD algorithm.

Supplementary Note 6: Far-field beam characterization setup for OPA.

Figure S8 shows the far-field characterization setup for the OPA. Since the OPA chip emits optical beams at a specific angle, a metal bracket is designed to mount the chip vertically. This ensures forward emission of the beams. The setup includes a laser, a PC, the OPA chip, a screen, an infrared camera, a host computer, a FPGA multichannel voltage source, and a rotating arm. Light from the laser first passes through the PC for polarization adjustment and is then coupled into the chip via an optical fiber. The infrared camera captures the far-field pattern projected by the OPA onto the screen and transmits the image to the host computer for display. The multichannel power module connects to the OPA PCB to supply electrical signals. After phase error calibration, the rotating camera captures the far-field distribution across the entire FOV.

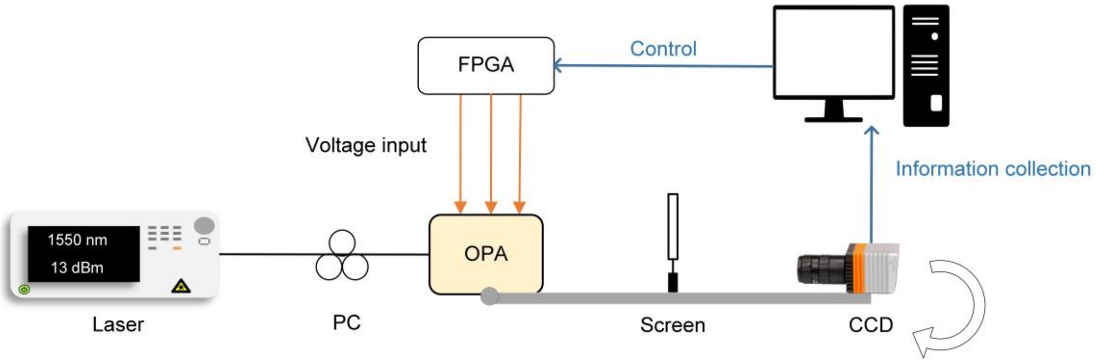

Figure S8 Far-field characterization setup for the OPA.

A key observation from this setup is the degradation of OPA spot quality. This occurs due to direct current (DC) drift when the optimized voltage is continuously applied. DC drift is an inherent property of TFLN modulators. It disrupts the coherent superposition of optical beams in each OPA channel under in-phase conditions. As a result, energy in the OPA beam spot diffuses, sidelobe intensity increases, and pointing accuracy decreases. To mitigate this issue, studies show that process optimization, such as removing the cladding structure of LN waveguides, can effectively suppress DC drift [6]. This strategy is essential for maintaining long-term stability of OPA beam performance. In addition to phase calibration and DC drift suppression, the setup also enables additional beam control functions. Wavelength-based angular steering can be achieved by adjusting the laser output wavelength. This capability enhances the flexibility of the OPA for beam steering applications, supporting its potential use in

high-performance optical communication systems.

Supplementary Note 7: Power consumption principle and calculation.

Beam steering via phase control in an OPA fundamentally relies on precise phase modulation at each emitter. The TFLN electro-optic phase shifter serves as a critical component for this modulation. Its operating mechanism directly determines the phase control accuracy and energy efficiency of the OPA. The TFLN electro-optic phase shifter has a capacitor-like structure. When a voltage is applied across its electrodes, the resulting electric field alters the refractive index of the TFLN layer. This modulates the phase of the optical wave propagating through it. Thus, each phase modulation unit can be modeled as a capacitor (*C*). Unlike conventional thermo-optic OPAs, whose power consumption is dominated by thermal dissipation, TFLN-OPA phase shifters utilize the Pockels electro-optic effect. This mechanism involves only the conversion of optical phase energy and does not produce Joule heating losses. The main power consumption in TFLN-OPA phase shifters arises from switching dissipation, which occurs during voltage switching operations when driving the capacitive load. The energy variation in the phase shifter is described by:

$$E=\frac{1}{2}CV_{rms}^{2} \tag{S28}$$

where C is the capacitance and V_{rms} is the root mean square (RMS) voltage.

To characterize the phase shifter, both experiments and simulations are performed. Using the setup in Figure 4a, the half-wave voltage of a phase modulator with an electrode length L of 8 mm is measured as 4.7 V (Figure 4b). The capacitance is simulated using ANSYS HFSS, yielding an average value of 0.79 pF. Based on the above equation, the power consumption per phase shifter is calculated to be 8.73 pJ/π. This low power consumption of 8.73 pJ/π represents a significant advantage of TFLN-OPAs over conventional thermo-optic OPAs. The latter typically exhibit much higher power consumption due to thermal dissipation, limiting their use in energy-sensitive applications such as integrated optical communication systems. The low energy requirement of TFLN-OPA phase shifters makes them highly suitable for high-density, low-power integrated optical devices.

Supplementary Note 8: OPA working distance and stability measurement device. To characterize the working distance and operational stability of the OPA, we design a dedicated measurement setup. Figure S9 shows the schematic diagram of the setup. The measurement system consists of a laser, a erbium-doped fiber amplifier (EDFA), a PC, the OPA, a beam collimation assembly, a collimator, and an optical power meter. The PC adjusts the polarization state of the laser beam before it enters the OPA. The OPA achieves specific beam deflection or scanning through internal phase control. Although the beam emitted from the OPA is divergent, the collimator receiver requires parallel light. Therefore, a beam collimation assembly, which consists of a plano-concave lens and a plano-convex lens, is placed between the OPA output and the collimator. An optical power meter is installed after the collimator to monitor and record the received optical power in real time. In practical use, the OPA operates bidirectionally. When functioning as a receiver, it can directly receive non-collimated beams without requiring a lens assembly.

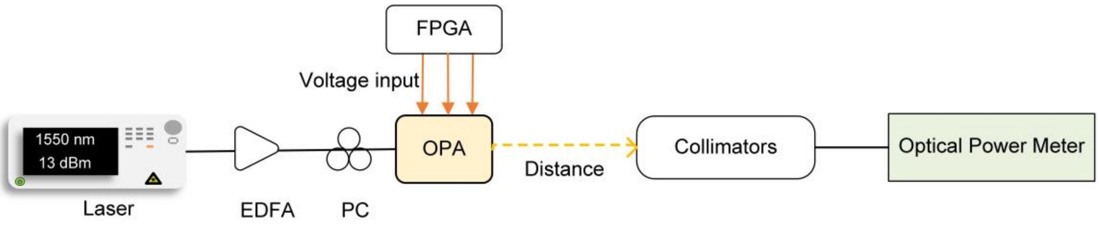

Figure S9 Schematic of the experimental setup used to characterize the OPA working distance and stability.

Measurements of the OPA's working distance aim to quantify its transmission performance over various propagation ranges. During testing, a precision displacement stage adjusts the relative distance between the OPA and the receiver collimator. This distance represents the free-space propagation length of the OPA beam before collimation by the lens pair. At each predefined distance, the received optical power is recorded only after both the system and the power meter readings stabilize. This procedure ensures that the measured values accurately represent the transmission capability of the OPA at each range.

The same experimental configuration is used to evaluate the operational stability of the OPA. This test examines how the OPA maintains performance over extended

periods. Throughout the stability measurement, the distance between the OPA and the receiver collimator remains constant, and all instruments operate under steady conditions. The optical power meter continuously tracks variations in received power, with data logged at one-minute intervals. This method captures slight power fluctuations over time, which is essential for verifying the OPA's reliability in long-term applications, such as continuous optical communication links. The working distance data inform the installation and deployment of the OPA in real-world systems, while the stability data confirm its suitability for scenarios demanding prolonged operation.

Supplementary Note 9: DSP process description for 320 Gbps 16-QAM transmission.

Figure 4b shows the digital signal processing (DSP) flowchart used in the 320 Gbps 16-Quadrature Amplitude Modulation (QAM) signal transmission experiment. At the transmitter side, the 16-QAM data stream is first upsampled. It is then processed using a root-raised cosine (RRC) filter with a roll-off factor of 0.01 for pulse shaping. The shaped QAM samples are sent to a digital-to-analog converter (DAC) operating at 100 GSa/s. The resulting electrical signals are amplified by two electrical amplifiers (EAs) before driving a 35 GHz IQ modulator biased at the null point. Meanwhile, a continuous-wave optical carrier at 10 dBm is pre-amplified with an EDFA and coupled into the IQ modulator. This modulated optical signal is further amplified by a second EDFA and finally transmitted through the OPA for OWC.

At the receiver, the system includes three core components: an EDFA for optical loss compensation, a coherent receiver for detection, and a 160 GSa/s digital storage oscilloscope (DSO) for data acquisition. The receiver DSP follows a step-by-step optimization procedure. First, the captured signal is resampled to remove sampling clock offset. Subsequent steps include root-raised cosine (RRC) matched filtering and symbol synchronization for signal preconditioning. A 2×2 multiple-input multiple-output feed-forward equalizer (MIMO-FFE) is used to correct linear impairments from the transmission channel. Since the FFE may amplify noise during equalization, an additional post-filter (PF) combined with maximum likelihood sequence detection (MLSD) is used to suppress noise and improve detection accuracy. Finally, the bit error rate (BER) is calculated and evaluated.

The curved interconnection waveguides, essential for pitch matching, introduce a maximum static path length difference of approximately 300 μm, corresponding to a time delay of about 2.2 ps. This delay is about 17.6% of a symbol duration for the 320 Gbps 16-QAM signal and therefore does not induce inter-symbol interference by overlapping adjacent symbols in time. Furthermore, the fixed phase offsets arising from these path differences are effectively calibrated out during the initial beam-forming process and are compensated by standard digital signal processing in the coherent

receiver, ensuring they have no measurable impact on communication performance.

Supplementary Note 10: Performance scaling of OPA.

Currently, most wavelength-tuned OPAs on TFLN platforms operate in the 1550 nm band. This operating range is constrained by the limited tuning range of available tunable lasers. For instance, when using a laser with a wavelength tuning range of about 100 nm, the achievable beam deflection angle in a wavelength-tuned OPA typically remains below 10°. This limitation restricts the number and capacity of communication channels that these devices can support. Existing TFLN-based OPAs primarily use transverse electric (TE) polarization. LN is optically anisotropic, allowing propagation and independent control of distinct polarization states. By utilizing alternative polarization states, particularly transverse magnetic (TM) polarization, the scanning range of OPAs can be significantly increased. Such improvement directly enhances communication capacity in OWC systems.

To investigate the effect of polarization on the far-field beam behavior at different wavelengths, we simulate the far-field patterns of the antenna under both TE and TM polarizations across multiple wavelengths. Figure S10a displays the resulting far-field profiles, illustrating the polarization-dependent beam steering behavior of the antenna. The profiles show clearly different steering angles for TE and TM polarizations at identical wavelengths, confirming that polarization state can serve as an independent control parameter for beam direction alongside wavelength tuning. To further evaluate the dual-polarization design, Figure S10b presents simulation results for the dual-polarization OPA-based antenna. Across the 1500–1600 nm wavelength range, a beam steering angle of up to 21.6° is achieved in the wavelength-sensitive direction through polarization control. This outcome shows that the dual-polarization strategy effectively extends the beam steering range compared to conventional single-TE-polarization OPAs, thereby mitigating the narrow scanning range typical of traditional designs.

To fully leverage this polarization-based enhancement, we design an antenna capable of simultaneously supporting two orthogonal polarization modes, TE and TM, with overlapping and complementary steering ranges. This configuration nearly doubles the wavelength steering range without requiring an expanded laser tuning range. It addresses a major limitation of conventional wavelength-tuned OPAs, where larger

deflection angles typically demand broader laser tuning. Consequently, this approach offers a practical solution for high-capacity OWC links. Notably, the design remains compatible with standard TFLN fabrication processes and commercial tunable lasers, eliminating the need for costly hardware modifications. This compatibility facilitates seamless integration into existing optical communication infrastructures, supporting the potential for broad practical deployment.

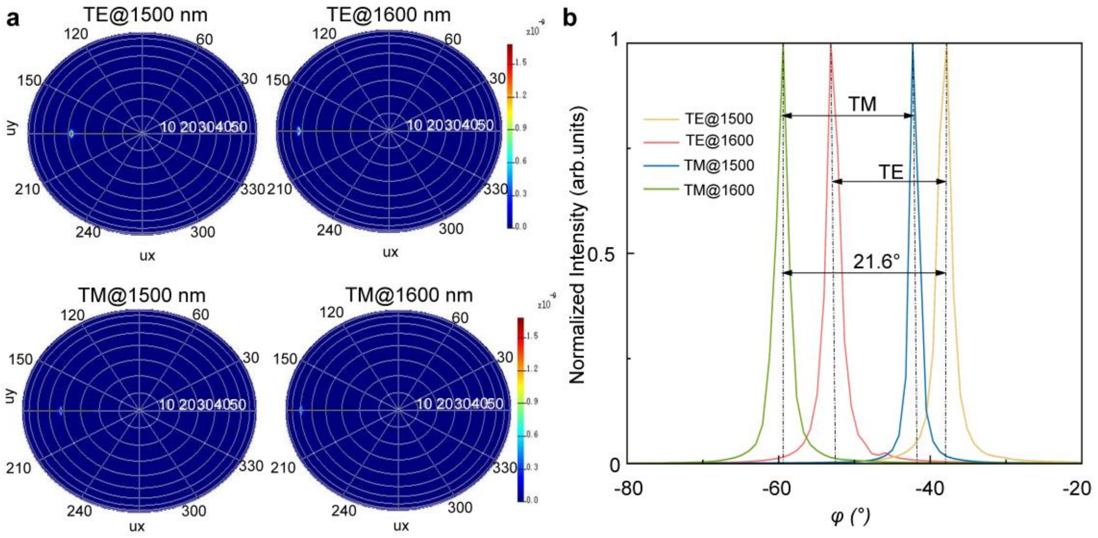

Figure S10 a Simulated far-field intensity distributions of the antenna for both TE and TM polarizations at the wavelengths of 1500 nm and 1600 nm. **b** The evolution of the far-field intensity distribution for TE and TM polarizations is presented. Simulated far-field intensity distribution of TE and TM polarization across the wavelength range from 1500 nm to 1600 nm.

Supplementary Table 1: TFLN OPA system performance comparison.

Table 1 TFLN OPA system performance comparison.

Representative works	Platform	FOV	Beamwidth	SLSR	Power consumption	Speed
Ref. 7	TFLN	50°×8.6°	0.73°×2.8°	-5.1 dB	330 pJ/π[b]	0.4 ns[b]
Ref. 8	TFLN	24°×8°	2°×0.6°	-10 dB	13.5 pJ/π[a]	0.23 ns[a]
Ref. 9	TFLN	62.2°×8.8°	2.4°×1.2°	-7.4 dB	1.11 nJ/π[b]	14.4 ns[b]
Ref. 10	TFLN	50°	8°	-10 dB	N/A	N/A
Ref. 11	TFLN-SiN	22°×5°	1.2°×0.2°	N/A	3.2 pJ/π[b]	0.83 ns[b]
Ref. 12	TFLN-SiN	62°×7.6°	3.2°×1.4°	-20.3 dB	41.6 pJ/π[b]	26 ns[b]
This Work	TFLN	62°×11°	3.8°×1.72°	-13.6 dB	8.73 pJ/π[a]	158 ps[b]

[a] Simulation results. [b] Actual experimental test results.

Supplementary References:

[1] Yu, L. et al. Adoption of large aperture chirped grating antennas in optical phase array for long distance ranging. *Opt. Express* **30**, 28112-28120 (2022).

[2] Huang, Y., Yu, C., Chen, C. Sidelobe-suppressed silicon waveguide gratings through asymmetrically-apodized corrugations. *IEEE Photonics T.* **36**, 629-632 (2024).

[3] Zhao, S., Chen, J., Shi, Y. Dual polarization and bi-directional silicon-photonic optical phased array with large scanning range. *IEEE Photonics J.* **14**, 6620905 (2022).

[4] Zhao, S. et al. Polarization multiplexing silicon photonic optical phased array with a wide scanning range. *Opt. Lett.* **48**, 6092-6095 (2023).

[5] Zhang, L. et al. Large-scale integrated multi-lines optical phased array chip. *IEEE Photonics J.* **12**, 6601208 (2020).

[6] Xu, Y. Shen, M. Lu, J. Surya, J. Sayem, A., Tang, H. Mitigating photorefractive effect in thin-film lithium niobate microring resonators. *Opt. Express* **29**, 5497–5504 (2021).

[7] Li, W. et al. High-speed 2D beam steering based on a thin-film lithium niobate optical phased array with a large field of view. *Photonics Res.* **11**, 1912-1918 (2023).

[8] Yue, G. Li, Y. Integrated lithium niobate optical phased array for two-dimensional beam steering. *Opt. Lett.* **48**, 3633-3636 (2023).

[9] Wang, Z. et al. Fast-speed and low-power-consumption optical phased array based on lithium niobate waveguides. *Nanophotonics* **13**, 2429-2436 (2024).

[10] Li, Y. et al. Design of optical phased array with low-sidelobe beam steering in thin film lithium niobate. *Optics & Laser Technology* **171**, 110432 (2024).

[11] Lee, W., Kwon, Y., Kim D., Sunwoo, Y., Lee, S. Hybrid integrated thin-film lithium niobate-silicon nitride electro-optical phased array incorporating silicon nitride grating antenna for two-dimensional beam steering. *Opt. Express* **32**, 9171-9183 (2024).

[12] Liu, J. et al. Taylor amplitude distribution for low sidelobe in thin-film lithium niobate optical phased array. *J. Lightwave Technol.* **43**, 8286-8292 (2025).
