# Supplementary material for: Multi-target and ultra-high-speed optical wireless communication using a thin-film lithium niobate optical phased array
Source: Nat Commun. 2025 Dec 15;17:969. doi: 10.1038/s41467-025-67696-3 (PMC12847847; doi:10.1038/s41467-025-67696-3)
Supplement: Supplementary file 2 — Description of Additional Supplementary Files [file 41467_2025_67696_MOESM2_ESM.pdf]

Description of Additional Supplementary Files

Supplementary Movie 1: Real time HD video transmission using the OWC system.

Supplementary Movie 2: The disconnection and reconnection processes of the OWC link.
