# Supplementary figures and images for: Multi-target and ultra-high-speed optical wireless communication using a thin-film lithium niobate optical phased array
Source: Nat Commun. 2025 Dec 15;17:969. doi: 10.1038/s41467-025-67696-3 (PMC12847847; doi:10.1038/s41467-025-67696-3)

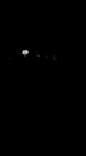

Supplement: Supplementary file 6 — Source data [file 41467_2025_67696_MOESM6_ESM.zip › Data/1550phase/-12.png]

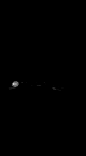

Supplement: Supplementary file 6 — Source data [file 41467_2025_67696_MOESM6_ESM.zip › Data/1550phase/-18.png]

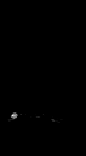

Supplement: Supplementary file 6 — Source data [file 41467_2025_67696_MOESM6_ESM.zip › Data/1550phase/-24.png]

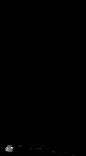

Supplement: Supplementary file 6 — Source data [file 41467_2025_67696_MOESM6_ESM.zip › Data/1550phase/-31.png]

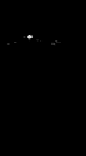

Supplement: Supplementary file 6 — Source data [file 41467_2025_67696_MOESM6_ESM.zip › Data/1550phase/-6.png]

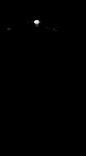

Supplement: Supplementary file 6 — Source data [file 41467_2025_67696_MOESM6_ESM.zip › Data/1550phase/0.png]

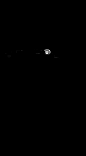

Supplement: Supplementary file 6 — Source data [file 41467_2025_67696_MOESM6_ESM.zip › Data/1550phase/12.png]

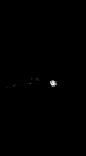

Supplement: Supplementary file 6 — Source data [file 41467_2025_67696_MOESM6_ESM.zip › Data/1550phase/18.png]

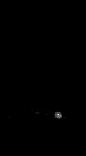

Supplement: Supplementary file 6 — Source data [file 41467_2025_67696_MOESM6_ESM.zip › Data/1550phase/24.png]

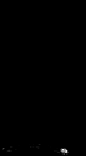

Supplement: Supplementary file 6 — Source data [file 41467_2025_67696_MOESM6_ESM.zip › Data/1550phase/31.png]

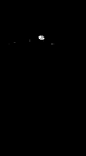

Supplement: Supplementary file 6 — Source data [file 41467_2025_67696_MOESM6_ESM.zip › Data/1550phase/6.png]

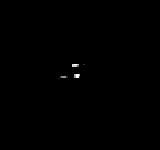

Supplement: Supplementary file 6 — Source data [file 41467_2025_67696_MOESM6_ESM.zip › Data/multi target/-10.png]

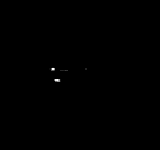

Supplement: Supplementary file 6 — Source data [file 41467_2025_67696_MOESM6_ESM.zip › Data/multi target/-20.png]

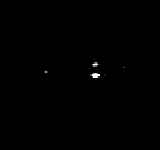

Supplement: Supplementary file 6 — Source data [file 41467_2025_67696_MOESM6_ESM.zip › Data/multi target/0.png]

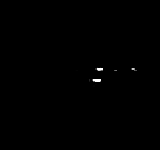

Supplement: Supplementary file 6 — Source data [file 41467_2025_67696_MOESM6_ESM.zip › Data/multi target/10.png]

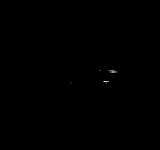

Supplement: Supplementary file 6 — Source data [file 41467_2025_67696_MOESM6_ESM.zip › Data/multi target/20.png]

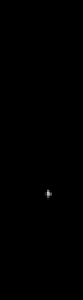

Supplement: Supplementary file 6 — Source data [file 41467_2025_67696_MOESM6_ESM.zip › Data/wavelength/1500.png]

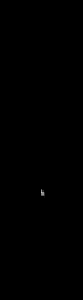

Supplement: Supplementary file 6 — Source data [file 41467_2025_67696_MOESM6_ESM.zip › Data/wavelength/1530.png]

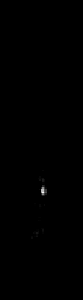

Supplement: Supplementary file 6 — Source data [file 41467_2025_67696_MOESM6_ESM.zip › Data/wavelength/1560.png]

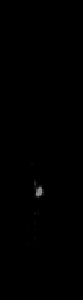

Supplement: Supplementary file 6 — Source data [file 41467_2025_67696_MOESM6_ESM.zip › Data/wavelength/1590.png]

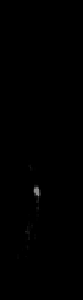

Supplement: Supplementary file 6 — Source data [file 41467_2025_67696_MOESM6_ESM.zip › Data/wavelength/1620.png]
